# MULTISENSOR APPROACHES FOR COMPREHENSIVE MEASUREMENT OF REAL-WORLD MOBILITY IN OLDER ADULTS

**DOI:** 10.1093/geroni/igae098.0743

**Published:** 2024-12-31

**Authors:** Karen Van Ooteghem

**Affiliations:** University of Waterloo, Waterloo, Ontario, Canada

## Abstract

Despite the potential for collecting precise and accurate mobility data over extended periods of time, the use of body-worn sensors as an approach to mobility monitoring is at a crossroads. The broad use of consumer smartwatches has led to a focus on single sensor approaches with simple to digest outcomes (e.g., step counts) however, this approach can limit our ability to capture details about how, where and when people are moving which are necessary to guide intervention. In this talk, we will provide evidence for the feasibility and value of a multi-sensor approach to mobility measurement that optimizes a’benefit-to-burden’ ratio for older adult participants. Specifically, we will provide examples to highlight how the use of multiple sensors can improve fidelity, reduce uncertainty, and provide important context to the data in order to maximize our understanding of real-life mobility. We will also share lessons learned from several multisensor-based studies of mobility across a spectrum of older adults (healthy, neurodegeneration, exceptional cognitive aging) that have contributed to successful implementation of a multi-sensor approach. The principles presented will inform future work aimed at developing validated standards for multi-sensor derived digital mobility outcomes that would provide comprehensive information on mobility for risk identification, early intervention, and monitoring of disease progression and treatment efficacy.

